# Long-Term Food Insecurity, Hunger and Risky Food Acquisition Practices: A Cross-Sectional Study of Food Charity Recipients in an Australian Capital City

**DOI:** 10.3390/ijerph16152749

**Published:** 2019-08-01

**Authors:** Christina M. Pollard, Sue Booth, Jonine Jancey, Bruce Mackintosh, Claire E. Pulker, Janine L. Wright, Andrea Begley, Sabrah Imtiaz, Claire Silic, S. Aqif Mukhtar, Martin Caraher, Joel Berg, Deborah A. Kerr

**Affiliations:** 1 Faculty of Health Science, School of Public Health, Curtin University GPO Box U1987, Perth Western 6845, Australia; 2 College of Medicine & Public Health, Flinders University, GPO Box 2100, Adelaide 5000, Australia; 3 School of Agriculture and Environment, The University of Western Australia, 35 Stirling Highway, Crawley, Perth 6009, Australia; 4 Centre for Food Policy, City University of London, Northampton Square, London EC1V 0HB, UK; 5 Hunger Free America, 50 Broad Street, Suite 1103, New York, NY 10004, USA

**Keywords:** food insecurity, hunger, social assistance, poverty, social security, charity, homeless, Australia

## Abstract

Inadequate social protection, stagnant wages, unemployment, and homelessness are associated with Australian household food insecurity. Little is known about the recipients of food charity and whether their needs are being met. This cross-sectional study of 101 food charity recipients in Perth, Western Australia, measured food security, weight status, sociodemographic characteristics and food acquisition practices. Seventy-nine percent were male, aged 21–79 years, 90% were unemployed, 87% received social assistance payments, and 38% were homeless. Ninety-one percent were food insecure, 80% with hunger, and 56% had gone a day or more without eating in the previous week. Fifty-seven percent had used food charity for ≥1 year, and, of those, 7.5 years was the mode. Charitable services were the main food source in the previous week, however 76% used multiple sources. Begging for money for food (36%), begging for food (32%), stealing food or beverages (34%), and taking food from bins (28%) was commonplace. The omnipresence and chronicity of food insecurity, reliance on social security payments, and risky food acquisition suggest that both the social protection and charitable food systems are failing. Urgent reforms are needed to address the determinants of food insecurity (e.g., increased social assistance payments, employment and housing support) and the adequacy, appropriateness and effectiveness of food charity.

## 1. Introduction

People facing uncertainty and risk in several areas of their lives are said to live in ‘precarious’ circumstances [1]. They are less resilient when faced with sudden events such as sickness, job or housing loss because they have limited reserves and a single event can cause a series of events over which they have little control. Social inequity and precarious living circumstances can result in health inequalities [2,3] including: smoking related conditions, cardiovascular disease, alcoholism and drug addiction, obesity, chronic infections, poor mental health and malnutrition [4,5]. Food insecurity, defined as the limited or uncertain availability of individuals’ and households’ physical, social and economic access to sufficient, safe, nutritious and culturally relevant food [6] is also associated with social and economic deprivation and other forms of adversity and can contribute to poor physical and mental health [7]. It is the role of Government and State institutions to protect people against precariousness or mitigate their experience of it, for example through social assistance payments [1]. Further, the impact of rising unemployment or stagnating wages on food insecurity is reduced in countries with robust social protection policies [8]. 

Fiscal policy to reduce poverty is considered good for health [9]. Australia is known for its wide-ranging and complex welfare safety net. The Australian minimum wage is nearly double the U.S. federal minimum wage (AUD$740.80 per week, which equates to a minimum hourly rate of AUD$19.49) [10], one reason why Australia has less food insecurity than the U.S., where one in ten working households are food insecure [11]. Yet there are increasing concerns regarding the adequacy and effectiveness of Australian social protection [12] and stagnating wages [13,14]. Even in Australia we see changes to welfare systems and the undermining of what has been achieved since the end of World War II (1945) and the acts for social insurance, what the French called ‘solidarité sociale’ [15]. For example, there has been a reduction in rates of entitlement, tightened means testing, and imposed obligation to work or look for work [16]. Recent austerity measures in some countries are contrary to the UN Committee on Economic, Social and Cultural Rights [15,17], specifically that they must be: a temporary measure for the period of crisis; that the policy is necessary and proportionate; that it is not discriminatory and comprise all possible measures including tax to support social transfers and mitigate inequalities; and finally, that they protect a minimum social protection floor [18].

In 2015–2016, 3.05 million Australians, (13.2% of the population) were estimated to live below the poverty line after taking into account housing costs [19] and, according to charitable food relief services, in 2018, over 710,000 people sought food relief each month [20]. Food charity, the delivery of donated, unsaleable, or waste food by the voluntary (non-profit) sector with limited government funding is the main response to food insecurity in Australia, despite the existence of the social protection system [21,22]. Government departments fund social service organisations to deliver emergency services, and it is estimated that 65% of funds are spent on food assistance [21]. In inner-City Perth, the charitable food system comprises three indirect services who source, bank and/or distribute food to 15 direct services who provide food to over 5670 people/week in 34 locations using 16 food service models [21].

Australia’s social security system consists of cash payments from the redistribution of Government taxation revenue and is intended to increase individual and family recipient’s wellbeing [23]. Social security and welfare expenditure in 2018–2019 was estimated at AUD$176.0 billion, or 36% of the Australian Government’s total expenses [24]. Payments include: income support pensions and allowances (for example, the Age Pension and Newstart Allowance), family payments such as Family Tax Benefit and Child Care Subsidy, Parental Leave Pay, funding for aged care services, the National Disability Insurance Scheme (NDIS), and payments and services for veterans and their dependents [24]. In addition, the government funds student allowances, and social insurance embedded into other systems (for example minimum wages, paid sick leave, and superannuation), and universal health care [16,24]. So, seemingly Australians should have a welfare safety net to protect them from food insecurity. 

Five million Australians relied on social security payments in 2018, half on the Age Pension or Veteran Service Pensions [25], 16% on a Disability Support Pension, 15% on Newstart (employment seekers payment for residents aged ≥22 years), 6.4% on Youth Allowance (available to full-time students and apprentices aged 16 to 24 years), and the remainder on Parenting Payment (available for parents with a child <6 years if partnered or <8 years if single, after which age parents must enter into a job plan), carer payments (available to persons providing constant care to one or more persons with a disability), and others [12]. 

There is an increased likelihood of food insecurity among people who are on social assistance payments in Australia, particularly the Newstart Allowance and the Disability Support Payment [26]. The Newstart Allowance has not increased since 1994, contributing to a progressive deepening of poverty for people where it is the main source of income, with 78% of Newstart Allowance recipients in poverty in 2015 [19]. Low income households spend proportionally more of their disposable income on food than those on an average or high income in Australia. Food affordability modelled as ‘food stress’ (which occurs when families need to spend over 25% of their disposable weekly income on food) [27], shows that households in receipt of welfare payments need to spent ~40% of their weekly disposable income on food to purchase a healthy diet compared to only 20% for average income households [28]. 

Approximately 4% of Australians are food insecure [29], with the prevalence higher among people who are homeless [30], Aboriginal and Torres Strait Islander people [29], refugees [31] and asylum seekers [32]. Australian population surveys have found that exposure to stressors related to employment and health approximately doubled the odds of experiencing food insecurity [33]. Single parent families in Victoria were 11 times more likely to be food insecure than couple-only families [34], and in Western Australia the odds of being food insecure increased 5.28 times in households with an income ≤AUD$20,000, and 5.29 times for 18 to 34 year olds compared to those aged >35 years [35].

Charities providing food assistance to food insecure people in Australia are concerned that they cannot meet increasing and chronic demand [20]. Individuals faced with a shortage of food cope in a variety of ways. U.S. research found many potentially risky food acquisition practices were used by people experiencing food insecurity, such as stealing, eating road kill or diluting infant formula [36]. Qualitative studies investigating recipient’s experiences and perceptions of Australian charitable food relief efforts consistently suggest that they get their food from multiple sources, rely heavily on the charitable sector, and that the food provided does not meet their needs [22,37,38,39,40,41,42]. However, very little is known about the food acquisition practices of individuals using Australian charitable food services. Service providers and policy makers are currently preoccupied with feeding people, above nutrition and health concerns, with little capacity to objectively measure the extent of service need, its appropriateness, or external factors impacting the current system. Understanding the breadth and nature of these food procurement practices can assist with developing appropriate responses to food insecurity [36]. This cross-sectional study investigates the food security status, sociodemographic characteristics, and food acquisition practices of a convenience sample of people accessing charitable food services located in inner-city Perth, Western Australia. 

## 2. Methods

### 2.1. Study Population and Recruitment

This cross-sectional study is part of research to assess the appropriateness and effectiveness of charitable food services operating in inner-city Perth, a precinct which has a population of 32,000 people residing in an area of 10.9 square kilometres [21]. Similar to other capital cities, homeless and transient individuals are drawn to the inner-city precinct where there is a concentration of food and other services. One service provider approached the authors concerned about the increasing demand for food assistance and questioning whether the service they provided was appropriate, particularly the suitability of serving hot meat pies as part of the soup van menu. As a result the researchers worked together with members in the sector to undertake a broad charitable food system project which considered the system, the services, models, and organizational capacity as well as also participant perspectives. This survey of recipients is one aspect of the research. Service providers in the area, along with those rescuing food were invited to participate in the research project. The sample of recipients of charitable food services located in inner-City Perth in January 2016 was recruited using flyers delivered by direct service staff or volunteers, word of mouth and posters displayed on soup vans. Interviewers attended field locations as volunteers for two weeks leading up to the survey date to familiarise themselves with the services and recipients. 

Participants were invited to attend a nominated charitable organisation during a three-hour period in the morning on three different days over two weeks. They were registered on arrival by a trained volunteer who was known to them, allocated an interview time, and offered a seat and a drink while they waited. The research information sheet was read by or to each person and verbal or written consent recorded. People with significant mental or cognitive impairment, limited English language or who were drug or alcohol affected were excluded from the study, as assessed by the trained volunteer who was familiar with the population. Curtin University Human Research Ethics Committee granted ethics approval (HR183–201) and AUD$10 cash was given at completion of the interview as compensation for their time and effort.

### 2.2. Measures and Instruments

A 38-item questionnaire, including both closed and six open-ended questions, was developed by the authors incorporating relevant questions from their previous research [43] with advice from a research advisory group comprising service providers and experienced researchers. The instrument collected: demographics (gender, age, educational attainment level, country of birth, ethnicity, living arrangements, and main source of income); health status (recent body weight changes and self-rated health and oral health); food acquisition habits (charitable food and related services usage, food sources, food purchasing behaviours, food preparation facilities, frequency of eating, and food they would buy if they had an extra AUD$20); assessment of charitable food services and access to other services. Household food insecurity experience was determined using the U.S. Department of Agriculture’s 6-item short questionnaire from Household Food Security Survey Module (HFSSM), with screeners (see questions HH3, 4 through to CH7) [44] chosen to reduce respondent burden. The questionnaire is available as a supplementary file.

The questionnaire was self-administered with offers of assistance if requested. A small number of participants were offered magnifying glasses to assist them to read. Upon completion of the written questionnaire, an interviewer reviewed the questionnaire with each interviewee to check for completeness and prompted them answer any missing questions. Participants were then invited to have their height and weight measured (fully clothed, without shoes) using a portable standing stadiometer and weighing scales.

### 2.3. Data Analysis

Responses to the written questionnaire, height and weight were entered into a custom-built database designed using Microsoft Access database platform. Data were audited for accuracy; inconsistencies were corrected with reference to the original data collection forms. Data was then entered into the SPSS version 24 (SPSS Inc., Chicago, IL, USA) for analyses. Frequencies were computed for demographic variables, and health/nutrition survey responses. Measured height and weight were used to derive Body Mass Index (BMI) classifications: underweight (BMI < 18.5 kg/m^2^); healthy weight (18.5–24.99 kg/m^2^); overweight (25–29.99 kg/m^2^); and obese (≥ 30 kg/m^2^) [45].

The food security status categories for the USDA HFSSM instrument were determined from a score calculated from the sum of the number of affirmative responses to the six questions about food security. Based on the summed score, participants were classified as food secure (0–1.99), low food security (2–4.99), and very low food security (5–6), and applying the affirmative responses to hunger: food secure without hunger; food insecure with hunger, moderate; and food insecure with hunger, severe [44].

## 3. Results

### 3.1. Participant Demographics

There were 101 participants who completed the study and their demographic characteristics are shown in Table 1. The majority were male (79%, *n* = 80), born in Australia (61%, *n* = 62), unemployed (90%, *n* = 90), and received social assistance payments (87%), with the Newstart Allowance and Disability support pension the most common. Their ages ranged from 22 to 79 years, with a mean age of 44.7 years (SD = 13.7 years). Almost half had completed high school and lived in single-person households. Thirty-four percent (*n* = 34) said that they had a fortnightly income of less than AUD$450 and 26% (*n* = 26) earned AUD$450–$549.

Participants had slept in 15 different types of accommodation at 194 different locations in the last month: sleeping rough i.e., in open air locations including the streets or in a park (*n* = 49), with friends (*n* = 26), in squats (*n* = 19), crisis accommodation (*n* = 15), private rentals (*n* = 15), own house (*n* = 11), and (*n* = 8) in shelters, backpackers, hostels and cars respectively, data not shown in Table.

### 3.2. Food Security Status and Going without Food

Overall, 91% (*n* = 88/97) of participants were classified as food insecure (80% (*n* = 78/97) severely food insecure with hunger, see Table 2. Three of the adult HFSSM questions included frequency of the characteristic over the last year and 35–63% of participants were experiencing these aspects of food insecurity almost every month or more frequently, 63% (*n* = 63/100) cut the size or skipped meals, 40% (*n* = 40/100) went to sleep at night feeling hungry, and 36% (*n* = 36/99) did not eat for a whole day because there wasn’t enough money for food. It was often true for 40% (*n* = 40/99) that the food bought didn’t last and they couldn’t afford more, and they couldn’t afford to eat balanced meals, see Table 2. 

In the past three months, 45% (*n* = 45/100) said they had lost weight, 32% (*n* = 32/100) said they did not know, 12% (*n* = 12/100) did not respond, and 9% (*n* = 9/100) said they had gained weight and 2/100 said they remained the same. Based on measured BMI, half (*n* = 50/100) were a healthy body weight, 34% (*n* = 34/100) were overweight, 16% (*n* = 16/100) were obese, and none were underweight. 

### 3.3. The Impact of Not Having Enough to Eat

Seventy-one percent (*n* = 71/100) of participants reported impacts on themselves and their family of not having enough food to eat on a regular basis. The impacts were classified as: negative physical impacts (feeling sick, weak, lethargic or tired, difficulty concentrating or sleeping, weight loss, starvation, overweight, and unmanaged medical conditions such as diabetes); emotional impacts (shame and depression, anxiety, worry, stress, mood swings, lack of motivation, and feeling unhealthy); and social impacts (relationship breakdowns, and reluctantly turning to crime for money or stealing food).

### 3.4. Food Acquisition, Preparation Facilities, Food Charity Use, and Preferences

When asked how much they usually spent on food each week, 67% (*n* = 67/101) spent AUD$21–100, 14% (*n* = 14/101) spent less than AUD$20, 11% (*n* = 11/101) spent over AUD$100, 7/101 spent nothing and 2/101 did not answer. Supermarkets (88% (*n* = 88/101) and take-away outlets (19% (*n* = 19/101)) were the main places they purchased food. Seventy-seven percent (*n* = 77/101) did not have a car for food shopping and 50% (*n* = 50/101) had no refrigerator, working stove or oven. Sixty-three percent (*n* = 53/100) said that food affordability was a problem and 73% (*n* = 73/101) did not have anyone to share food costs with. Sixty-six percent (*n* = 66/101) felt stressed because they could not afford to buy enough food and 32% (*n* = 32/101) said they had special dietary needs and that the foods they required were too expensive. 

Fifty-seven percent (*n* = 57/101) of participants had used charitable food services for a year or more, and, of those, 7.5 years was the modal length of time. Most (88% (*n* = 88/101)) said they did not have enough money in their budget to buy the food they needed and that they relied on others to either provide food or money for food when they ran out. 

When asked where they got food from in the previous week, charitable food services were the main source used by 76% (*n* = 77/101), followed by supermarkets (65%, *n* = 66/101), church or other welfare organisations (46%, *n* = 46/101), take-away outlets (32%, *n* = 32/101), food discarded from bins outside supermarkets or restaurants (19%, *n* = 19/101), and only 5% (*n* = 5/101) had sourced food from family or friends. More generally, participants reported that they had ‘sometimes or often’ begged for money for food (36%, *n* = 35/98), stolen food or drink (34%, *n* = 34/99), begged on the street for food (32%, *n* = 30/94), stolen money for food (19%, *n* = 18/94), or taken food from rubbish bins (28%, *n* = 27/94). When if they had gone without eating food for a whole day in the last week, 56% (*n* = 57/101) said yes.

Fifty-six percent of participants said they could not get the right quality of food and could not access the variety of food they would like. When asked which types of foods they would like to eat, recipients mentioned 310 different types of foods including: fruits and vegetables (30%), meat (27%), breads and cereals (21%), and meals (21%) such as salads, soups, roasts, spaghetti, barbeque, curry, casserole, sandwiches or ‘healthy’ food were the most sought after. When asked what they would buy if they had an extra AUD$20 to spend on food, participants listed 253 individual foods with meat (41%), fruit and vegetables (25%) and dairy foods (10%) the most common. 

Vegetarianism, using shelf-stable food items, and eating “*whatever is cheap and easy to make*” were some of the ways participant ensured they got to eat, but for less money. Only 28% said they needed to know more about how to make healthy meals. 

### 3.5. Recommendations for Charitable Food Services Improvements

Participants said it was important that services provide healthy food (93%, *n* = 91/97), cooked meals (84%, *n* = 82/98), water (82%, *n* = 80/98), knives, forks, and spoons (78%, *n* = 76/97), food and drinks served at the right temperature (76%, *n* = 75/98), coffee and tea (62%, *n* = 60/96), meat pies (56%, *n* = 54/96) and a place to sit and eat with others (53%, *n* = 52/98). When asked about ways to improve food assistance, participants suggested: more variety of food (more vegetables, protein foods and basic foods); up-to-date information advertising the times and location of services; longer opening hours and public holiday access; places to sit down and eat; facilities to cook their own meals; more funding for food services; incentives for people to become involved with charities; and links to additional services such as drug and alcohol programs.

### 3.6. Non-Food Service Utilisation

The majority of participants had utilised other services besides food assistance. The services most commonly accessed included general health services (64%, *n* = 63/99), employment assistance (59%, *n* = 58/98), accommodation assistance (58%, *n* = 57/99), and transport assistance (42%, *n* = 42/99). Less common were drug and alcohol services (29%, *n* = 29/99), reskilling or access to education services (29%, *n* = 29/99), and money management or budgeting services (25%, *n* = 25/99). 

## 4. Discussion

This study of people accessing charitable food services located in inner-city Perth found that despite acquiring food from multiple sources, 92% were food insecure and 80% were severely food-insecure with hunger. The high proportion of food insecurity among recipients of food relief services is to be expected and similar to findings reported in 2012 by one not for profit (Anglicare) who found that 96% were food insecure and 76% experienced severe food insecurity [46]. Further, over the last year 35–63% of participants in the current study had experienced aspects of food insecurity monthly or more often, 18% went to sleep hungry on a daily basis, and 45% had lost weight in the last three months. These results are particularly concerning because about half the sample had accessed food assistance for a year or more and still they are experiencing the most extreme form of food insecurity. 

The current study findings also quantify the precariousness and complexity of recipient’s social and living conditions, with many on an inadequate income, experiencing unemployment, and lacking permanent accommodation. The study sample included people with disabilities, experiencing unemployment or underemployment as well as 10% who were employed, and 87% were recipients of welfare payments. Western Australian Government population surveillance surveys also show a higher prevalence of food insecurity among people who are: unable to work (18%); the unemployed (13%); and, holders of a low income Health Care Card, concession cards for cheaper healthcare or pharmaceutical discounts (11%) [35]. 

This research is the first Australian study that quantifies the long-term reliance on food assistance, 7.5 years for those using services over a year. The chronic and severe food insecurity identified in the current study challenges the Australian food relief system which was established over a century ago to provide supplementary food in times of need; but defines that need as temporary ‘emergency food relief’, usually for a few days at a time [22]. Clearly, the need is chronic and is related to the persistent underlying social and economic circumstances. Overall, the findings suggest an ineffective and inadequate charitable food service system and Government welfare system.

Most of the recipients had very low reported income levels; 67% were relying on ≤AUD$549/fortnight, compared to the adult average fortnightly earnings of AUD$2500 in 2018 in Australia [47]. Two-thirds of the current study sample were surviving on incomes that were much less than the income reported by participants in other relevant studies. An Australia-wide survey of food insecure households in 2012 found 67% had incomes less than $1000/fortnight [46] and only 9% of 18 to 64 year old persons accessing mental health services in the Hunter Valley region earned less than AUD$500/fortnight [48]. The majority (87%) of the current study recipients were in receipt of social security payments, similar to other relevant surveys (89% of the Hunter Valley sample [48] and 75% of the 2012 Anglicare sample) [46]. The need to seek food assistance in the current study suggests that social security payments are inadequate, and, when compared to the proportion of people accessing social welfare payments in 2012, the situation has worsened over time. 

Social protection throughout life and universal health care can be social determinants of health for some groups [49], and can protect those people facing economic hardship from food insecurity, but greater protection may be required at these times [8]. Social protection is a human right and is intended to provide an adequate income to allow people to live a socially-fulfilling life. Recent policy reforms tightening the eligibility of payments and requiring mandatory participation (e.g., mandating that a minimum of 15 h per week be spent looking for paid employment, engaged in work, training, or study) have shifted many people on the Parenting Payment Allowance to the lower value Newstart Allowance [12]. Research examining the health and wellbeing impact of these decreased payments for single parents in Australia found that their food practices had changed as a result [50]. 

Our findings resonate with social services’ advocacy calls for nutrition-sensitive strategies such as increasing welfare payments, particularly for the third of participants in receipt of Newstart Allowance and the 22% receiving Disability Support Payments [19]. Australian Government increases in social security and welfare expenditure of 26.9% between 2018 and 2019 were in response to the ageing population and the roll out of the new National Disability Insurance Scheme (NDIS). Planned increases in 2021–22 are for AUD$15.8 billion for the NDIS, AUD$8.7 billion for Age Pension and other income supports for seniors, AUD$5.5 billion for Aged care services, and only AUD$0.8 billion for the Newstart Allowance and Youth Allowance [24]. Implicit in these planned increased is the distinction between the deserving and the undeserving poor.

One in ten of those seeking food assistance in the current study were employed, reinforcing concern regarding wage stagnation, under-employment and casualisation of the Australian workforce [13,14]. In 2018, 53% of Australians living below the poverty line relied on social security as their main source of income and 38% were employed [19], reinforcing the concern about the inadequacy of wages. Clearly, it is not only those relying on welfare who experience food insecurity in Australia. Employment status remains strongly associated with the severity of food insecurity [51]. Analysis of recent Australian government population health surveillance surveys also shows food insecurity prevalence is increasing among wage earners [34,35]. 

The majority of participants reported numerous negative physical, emotional and social impacts of long-term food insecurity on themselves and their families. Hecht et al. (2018) asserts that experiencing food insecurity itself creates trauma (an emotionally painful, distressing or shocking experience) and can result in risky behaviour such as stealing, which in turn increases the potential for further trauma [7]. Despite the heavy reliance on food assistance among participants in the current study some people had resorted to risky and potentially traumatic food acquisition practices (e.g., stealing or begging for money for food, stealing food, or taking food from rubbish bins). Risky food acquisition practices were undertaken by ~30% in the current study and up to 65% in a 2003 Adelaide study of homeless youth [43], higher than the 5% reported by US food pantry users [36]. This further supports the argument that food assistance, welfare income, and/or minimum wages are inadequate in Australia. Other reasons people resort to risky food acquisition include: the strict and stigmatising food relief eligibility assessment; the short-term nature of food relief; unsuitable types and amounts of food offered; undignified food service models; no choice; monotony of the food supplied; and the lack of advertising and information regarding food assistance [37,42]. 

Risky food acquisition practices may cause stress and shame because these activities lie outside the boundary of social and cultural norms [36,38]. These practices can increase financial, health (e.g., nutritional, foodborne illness), legal (fines for stealing, trespassing), and/or physical risk [36]. The consequence of theft, begging and stealing food can be prosecution and fines [52]. Laws related to stealing food in Australia aim to protect individuals from foodborne illness and retailers from liability. The consequences of stealing food may be outweighed by the need for food and preferable to the stigma experienced accessing food charity [38,53,54]. 

Food recipients’ accommodation situations varied, 60% had some form of accommodation, either renting (38%), mortgage (5%) or hostel (17%), and 38% were homeless. Unstable housing and accommodation and lack of washing, sanitation, cooking and food storage facilities are symptomatic of poor housing and/or the unaffordability and insecurity of tenure [55]. Scant attention has been paid to unhealthy housing in Australia due to the mild climate and because housing stock is in relatively good condition [56]. However, the unhealthiest housing tends to be located in major urban centres, reflecting high housing costs and limited affordability. The limited access to food preparation facilities in the current study is likely to be due to: being non-domiciled; financial circumstance prohibiting purchases of whitegoods; inability to pay for utilities; and, no food storage or cooking facilities at accommodation. 

People who are homeless find access to food a major issue with food insecurity a feature of their daily lives [57]. Homelessness is known to be associated with food insecurity, poorer physical and mental health and increased mortality [58,59]. In Australia, national homeless statistics show long-term homelessness with almost three quarters of youth and over half of adults homeless for over six months. It is estimated that 10,000 people are homeless in Western Australia, but the number in Perth is unknown [60]. Case studies of the total cost to the healthcare system based on frequent emergency department presentations and hospital admissions by three homeless individuals in Perth was ~AUD$333,000, ~AUD$250,000, and ~AUD$623,000 over 2–2.5 years [3].

Emergency relief assistance can include help with purchasing food, utility bills, no interest loans, financial counselling, and support with affordable housing [46]. Seeking health and social services may be difficult due to addiction, lack of transport, and perceived stigma when seeking help from agencies [59]. The findings suggest there is an opportunity to increase usage of services to assist pathways out of poverty and hardship. About half of the participants in the current study had accessed health services, employment, housing and transport assistance and less than a third had used drug and alcohol reskilling or access to education, or money management or budgeting services. Food relief is often the first point of contact for people experiencing hardship and offering ‘more than food’ in a dignified setting makes sense in terms of addressing immediate and long term need [61].

A comprehensive range of strategies is needed to prevent and address food insecurity. Community kitchens aim to improve food security, cooking skills, nutrition and to reduce social isolation. Although they may improve social interaction and nutritional intake, rigorous impact evaluation is required before recommending them as an evidence-based public health intervention [62]. Attention needs to shift to the underlying drivers of social inequities as an important part of the solution to improving dietary inequities [63]. ‘Nutrition-sensitive’ policies, social and economic policies implemented outside the health system, are recommended for their wider and long-lasting impact [63]. Social protection interventions, both cash transfers and food subsidies, reduce household food insecurity, whereas community-level interventions, such as food banks and other food programmes, have limited impacts [64]. The current study is consistent with the finding that recipients of social security payments have higher prevalence of food insecurity in Australia, reinforcing the need for ‘up stream’ policy to address food insecurity [26]. There is also a need for ‘nutrition-specific’ policies which aim to directly influence and improve the food supply and consumption for high risk groups.

This research was conducted as a result of questions raised by volunteers and recipients of charitable food services in inner-city Perth in 2014, about the ongoing capacity of services to meet the increasing demand for food relief. Preliminary research revealed that recipients were grateful for food assistance but were also negatively impacted by it [21,37]. Initial interviews suggested that many of the deadly “*ins*” of food charity described by Janet Poppendieck in 1999 and McIntyre et al. in 2016 [65,66] existed, including that the food provided by services was inappropriate, inadequate and insufficient, and that the services were sometimes inaccessible, inappropriate, and inefficient. The impact of these deadly “*ins*” is even more significant given the current study finding that for 75% of recipients, food obtained from charitable food services was the main source of food in the past week. 

A previous assessment of the organisational capacity of charitable food services in inner-City Perth also revealed the precarious circumstances under which they operate, due to unreliable, insufficient and inappropriate financial, human and food resources and structures [21]. The charitable food system itself demonstrated all four of Salamon’s (1987) voluntary failures including: ‘philanthropic: insufficiency’, the “*inability to generate resources on a scale that is both adequate enough and reliable enough to cope with the human-service problems*” pg 39, particularism, “*the tendency of voluntary organizations and their benefactors to focus on particular subgroups of the population*,” pg 40, paternalism, the “*approach inevitably vests most of the influence over the definition of community needs in the hands of those in command of the greatest resources*,” pg 41, and amateurism, “*association with amateur approaches to coping with human problems*” pg 42 [67]. 

The findings suggest three significant failures [68]: firstly, an Australian market failure, where private interests have not efficiently and fairly distributed society’s goods; in this case food is the private good, and food security, where all individuals in the society benefit from this condition, is the public good. Secondly, government failure in not providing leadership, policy guidance or directly providing adequate or appropriate food to services who rely almost entirely on the voluntary sector. Thirdly, a voluntary failure, as the system developed to meet the needs of emergency short-term food relief clearly fails to meet the current and increasing needs of vulnerable members of society. The Australia charitable food system provides temporary ‘emergency food relief’, usually for a few days at a time [22]. Collectively these failures have contributed to the rise, proliferation and corporatisation of a sophisticated foodbanking system operating in parallel to the dominant food system and delivering donated surplus or waste food to frontline food charities [15,69,70]. Foodbanks have been described as the de facto welfare safety net in the UK [71]. 

The weight of evidence suggests that adequate welfare is a means of lifting families out of poverty, especially when health and education services are included [15,72,73]. Yet Australian foodbanks and food rescue organisations are prioritised as the key response in the government’s reporting against the Sustainable Development Goal’s (SDG) implementation (poverty (SDG1), hunger (SDG2) and sustainable consumption and production (SDG12)) [74]. Despite the efforts of these not for profit organisations, the current findings show that people rendered vulnerable due to their social and economic circumstances remain chronically food insecure in Australia. 

The charitable food system in Australia is accepted as ‘normal’ practice in part because it has been operating this way for over 200 years [22]. The cultural normalisation of food charity can inadvertently normalise poverty and legitimise personal generosity or food waste, as an answer to major social, political and economic problems, over strong government leadership and intervention [75]. The current focus on trying to meet the unmet demand by improving food supply chain logistics fails to acknowledge the causes and chronicity of food insecurity and will further embed food charity as the main response. The unintended consequence of this may in fact be a reduction in human rights related to food. The value of reducing food insecurity by implementing nutrition-sensitive responses (eg. addressing unemployment, inadequate income, poor housing, and social disadvantage) is not part of the current dialogue, but is urgently needed. Some form of emergency food relief will always be required in response to unpredictable circumstances. To be effective, emergency response should provide food that is adequate, appropriate, nutritious and accessible in a non-stigmatising way, and support recipients into pathways out of food insecurity. A comprehensive range of evidence-informed strategies that are both nutrition-specific and nutrition-sensitive are needed to reduce the prevalence and impact of food insecurity. 

### Strengths, Limitations and Further Research

A strength of the study was the Research Advisory Committee guidance providing emergency relief and health services, university, users and local government perspectives. Their local knowledge and experience assisted with all aspects of the survey design, implementation and interpretation of findings. The Committee’s advice ensured the research protocol was client-centred. The structured training instrument administration and daily debriefing further enhanced the results.

The methodology was successful in recruiting a hard-to-reach and extremely vulnerable population, and, despite the time commitment of 60–90 min, only one participant did not complete all parts of the research. The high levels of participation and recruitment were likely due to: some researchers being known to participants (e.g., BM & JP were volunteers at the services); interviews being held in well-known, secure and safe premises at emergency services that were familiar and comfortable for participants; snacks and drinks provided to assist participant concentration; and referral to additional services if needed. 

The convenience sample of people attending charitable food services in inner-city Perth recruited for this survey may not be representative of all users in Perth or Australia, or reflective of recipients in other countries. The total number of people using these services in Australia is unknown and, as people only use charitable food services as a last resort, it is likely that some groups are under-represented [38], e.g., the working poor. There were 310 street-present people (i.e., people who spend time wandering the streets for a substantial proportion of their day or night) identified in an audit conducted in inner-City Perth just prior to the current study [76]. Therefore, the sample size of 101 recipients of food assistance in the same area is likely to include a high proportion of those on the street. Given how hard to reach this population is, these findings are important. Australian services do not currently monitor the number of people seeking food relief, the number they provide food to, or the length of time people receive food for. Rather, food relief performance is measured against the number of meals provided and the kilograms of food distributed.

Although the study measured the length of time participants’ had utilised food relief services, a limitation is that the questions did not allow the distinction between prolonged, persistent and frequent users and future research should differentiate these. This information is crucial to tailoring effective services to meet client needs. Kaiser and Cafer developed a suitable typology to categorise households accessing pantry service in the US: ‘persistent users’ are those who access services for more 24 months (longevity) and at least once a month (regularity) over a 12 month period, whereas prolonged users are long-term but infrequent users, less than monthly over the last 12 months [77]. 

The ethics proposal for this research recognised the potential impact of working with vulnerable populations on both the researcher and the participant. The research plan outlined precautions such as providing participants with a safe, monitored environment, magnifying reading glasses, chairs and tables, and refreshments at the time of the interviews. Despite considering the vulnerability of the recipients and anticipating participant’s needs, the researchers themselves were not fully prepared for the emotional impact of undertaking the interviews. The level of unprompted disclosure of personal trauma and experience, as well as the poor physical health of interviewees, was distressing. For example, during interviews some participants expressed sadness and said they felt ashamed of the predicament they found themselves in, others were so hungry that interviews were interrupted to provide food. Many of the interviewees arrived carrying all of their worldly belongings, usually in one large bag or shopping trolley. 

This current research reinforces the need for and importance of thorough preparation to avoid unintended consequences. Some researcher-participant interactions can expose researchers to levels of distressing or traumatic details, and unfamiliar circumstances [78]. As a consequence of observing degrees of human suffering first-hand and reflecting on their own privileged positions, even experienced researchers may be emotionally impacted - and indeed this occurred in our study. The experience of conducting the interviews reinforces the value of ethical protocol in the identification of risks to vulnerable populations, for example, considering physical needs of participants, ensuring sufficient time for and between interviews, and scheduling researcher de-briefings [78]. 

The current study is part of a broader research project which aimed to assess the charitable food sector and needs of those it services in inner-city Perth. The research has three main arms: an organizational mapping to describe the players, relationships, capacity and key functions [21], direct service’s and recipients’ perspectives on the appropriateness and effectiveness of current charitable food services [37], and, finally, recipients’ food procurement practices (this current study) and dietary intakes. The findings from all parts of the research project will be used to guide policy and program planning.

## 5. Conclusions

The omnipresence of severe and chronic food insecurity and the use of risky food acquisition practices among recipients of food assistance in inner-city Perth in this study demonstrates the failure of the current system to adequately meet their food needs. Recipients thought healthy meals provided in dignified settings (at the correct temperature with knives and forks etc.) were important. The high reliance on social security payments suggests that both the charitable food system and the social security systems are failing to meet the needs of vulnerable people. Findings add weight to calls to reform the Australian response to food insecurity. Both nutrition-sensitive and nutrition-specific policy options are needed to give people pathways out of food insecurity by providing sufficient money to meet the requirements of daily living, and to develop appropriate, effective and efficient food assistance where necessary. 

## Figures and Tables

**Table 1:** Demographic characteristics of direct service recipients’ of Charitable Food Services in Inner City Perth, Western Australia (WA), January 2016 (*n* = 101).

Characteristic		Count	%
Sex	Male	80	79.2
	Female	20	19.8
	Transgender	1	1.0
Age (years)	21–30	17	16.8
	31–40	24	23.8
	41–50	27	26.7
	51–60	18	17.8
	61–70	7	6.9
	71–80	6	5.9
	Refused	2	2.0
Aboriginal	Yes	20	19.8
	No	81	80.2
Education	Primary school	6	5.9
	High school	49	48.5
	College/TAFE	32	31.7
	University	14	13.9
Country of Birth	Australia	62	61.4
	UK/European	20	19.8
	New Zealand	8	7.9
	Asia	7	6.9
	Other/refused	4	4.0
Income (AUD$/fortnight)	0–249	11	10.9
	250–449	23	22.8
	450–649	34	33.7
	650–1000	19	18.8
	Refused	14	13.9
Main income source ^1^	Social security	84	87.4
	Income or Waged	11	10.9
	No Income	5	5.0
	Family/friend/partner	1	1.0
Social security income	Newstart	37	36.6
	Disability Sickness	27	26.7
	Partner allowance	1	1.0
	Youth Allowance	13	12.9
	Age Pension	6	5.9
Accommodation	Rent	38	37.6
	Live on the street	38	37.6
	Hostel	18	17.8
	Own home/mortgage	5	5
	Refused	2	2

^1^ 8 participants reported a second income source: 4 family and friends; 2 income/wages; 2 Newstart.

**Table 2:** Factors related to food insecurity among recipients of Charitable Food Services in Inner City Perth Western Australia (WA), January 2016 (*n* = 101).

Food Security Status and Components	Categories	Count	%
Food security status in the last 12 months, *n* = 97	Food secure	8	8.3
	Food insecure without hunger	11	11.3
	Food insecure with hunger	78	80.4
Food security components in the last 12 months:			
…did you ever cut the size of your meals or skip meals because there wasn’t enough money for food?, *n* = 100	Almost every month	63	63
	Some months but not every month	17	17
	Only 1 or 2 months	6	6
	No	14	14
…did you ever eat less than you felt you should because there wasn’t enough money for food?, *n* = 99	Yes	81	82.7
	No	14	14.3
	Don’t know	3	3.1
…were you every hungry but didn’t eat because there wasn’t enough money for food?, *n* = 100	Yes	79	79
	No	20	20
	Don’t know	1	1
…were there any times that you ran out of food and you couldn’t afford to buy more?, *n* = 100	Yes	88	88
	No	12	12
…did you ever not eat for a whole day because there wasn’t enough money for food?, *n* = 99	Yes, almost every month	36	36.4
	Yes, some months but not every month	22	22.2
	Don’t know	12	12.1
	No	29	29.3
…the food that I/we bought just didn’t last, and I/we didn’t have money to get more., *n* = 99	Often true	40	40.4
	Sometimes true	48	48.5
	Never true	10	10.1
	Don’t know	1	1
…I/we couldn’t afford to eat balanced meals., *n* = 101	Often true	44	43.6
	Sometimes true	44	43.6
	Never true	6	5.9
	Don’t know	7	6.9
…did you go to sleep at night feeling hungry?, *n* = 100	Almost every day	18	18
	Almost every week	17	17
	Almost every month	5	5
	Some but not every month	23	23
	Only 1 or 2 months	12	12
	No	25	25

## Supplementary Materials

The following are available online at https://www.mdpi.com/1660-4601/16/15/2749/s1.
