# Supplementary material for: Long-Term Food Insecurity, Hunger and Risky Food Acquisition Practices: A Cross-Sectional Study of Food Charity Recipients in an Australian Capital City
Source: Int J Environ Res Public Health. 2019 Aug 1;16(15):2749. doi: 10.3390/ijerph16152749 (PMC6696626; doi:10.3390/ijerph16152749)
Supplement: Supplementary file 1 [file ijerph-16-02749-s001.pdf]

ID

food survey

The School of Public Health at Curtin University Food Survey aims to find ways to improve the food service on the street. Thank you for agreeing to participate, we will take about an hour of your time.

There are three parts to this study. You will be asked to:

1. have your height and weight measured,
2. complete a record of the food you ate yesterday, and
3. fill out this questionnaire.

This information and all your answers will be confidential and you can stop at any time. If you don't want to answer a question, just leave it blank. If you don't understand a question please check with me.

If you have any questions about the study or your involvement, please ask me in person or telephone Dr Christina Pollard on 08 9266 1142.

Name you like to be called by :……………………………………………….

Age: ……………… (years) Gender: ☐ Female ☐ Male

Country you were born in? …………………………………………………..

What is your highest level of education?

☐ Primary School ☐ High school ☐ College or TAFE ☐ University

Do you identify as Aboriginal or Torres Strait Islander? ☐ Yes ☐ No

Curtin University Human Research Ethics Committee approval number HR183/2015

1. In the last 12 months, since January last year, did you ever cut the size of your meals or skip meals because there wasn't enough money for food?
   - ☐ Yes, almost every month
   - ☐ Yes, some months but not every month
   - ☐ Yes, only 1 or 2 months
   - ☐ No

2. In the last 12 months did you ever eat less than you felt you should because there wasn't enough money for food?
   - ☐ Yes
   - ☐ No
   - ☐ Don't know

3. In the last 12 months were you every hungry but didn't eat because there wasn't enough money for food?
   - ☐ Yes
   - ☐ No
   - ☐ Don't know

4. In the last 12 months were there any times that you ran out of food and you couldn't afford to buy more?
   - ☐ Yes
   - ☐ No
   - ☐ Don't know

5. In the last 12 months, did you ever not eat for a whole day because there wasn't enough money for food?
   - ☐ Yes, almost every month
   - ☐ Yes, some months but not every month
   - ☐ Yes, only 1 or 2 months
   - ☐ No

People have made statements about their food situations. How often do these apply to you?

6. In the last 12 months, the food that I/we bought just didn't last, and I/we didn't have money to get more.
   - ☐ Often true
   - ☐ Sometimes true
   - ☐ Never true
   - ☐ Don't know

7. I/we couldn’t afford to eat balanced meals.
   - ☐ Often true
   - ☐ Sometimes true
   - ☐ Never true
   - ☐ Don’t know

8. Do any children, family or others rely on you for food and shelter?
   - ☐ No, **go to question 11**
   - ☐ Yes, please describe their relationship to you and their age

…………………………………………………………………………………

…………………………………………………………………………………

9. In the last 12 months, I/we couldn’t feed my/our children a balanced meal, because I/we couldn’t afford that.
   - ☐ Often true
   - ☐ Sometimes true
   - ☐ Never true
   - ☐ Don’t know

10. In the last 12 months, my/our children were not eating enough because I/we just couldn't afford enough food.
   - ☐ Often true
   - ☐ Sometimes true
   - ☐ Never true
   - ☐ Don’t know

11. Which of the following statements are true for you?

	True	False
I can’t get food of the right quality	☐	☐
I can’t get a variety of food	☐	☐
I don’t have enough money in my budget to buy food I need	☐	☐
I rely on others to provide food or money when I run out of food	☐	☐
I feel stressed because I can’t afford enough food	☐	☐
I don’t have a car for shopping	☐	☐
I don’t have a fridge	☐	☐
I don’t have a working stove or oven	☐	☐
I need to know more about making healthy meals	☐	☐
I don’t have anyone to share food costs with	☐	☐
I have special dietary needs but these foods are too expensive	☐	☐
I should eat more fresh foods but they are too expensive	☐	☐
I have health issues	☐	☐

12. How many times a day do you usually eat?
    - ☐ I don't eat every day
    - ☐ Once a day
    - ☐ 2-3 times a day
    - ☐ 4 or more times a day

13. Last week, did you go a whole day or more without eating anything?
    - ☐ Yes If yes, how many days _________
    - ☐ No

14. In the l2 months, did you ever go without eating anything for more than one day?
    - ☐ Yes, almost every week
    - ☐ Yes, almost every month
    - ☐ Yes, some months but not every month
    - ☐ Yes, only 1 or 2 months
    - ☐ No

15. In the l2 months, did you go to sleep at night feeling hungry? (tick one box)
    - ☐ Yes, almost every day
    - ☐ Yes, almost every week
    - ☐ Yes, almost every month
    - ☐ Yes, some months but not every month
    - ☐ Yes, only 1 or 2 months
    - ☐ No

16. What are the main kinds of foods you want to eat?

………………………………………………………………………………………

………………………………………………………………………………………

………………………………………………………………………………………

17. Do you find it hard to get the kind of foods you want to eat? (tick one box)
    - ☐ No
    - ☐ Yes, please explain……………………………………………………..

………………………………………………………………………………………

………………………………………………………………………………………

………………………………………………………………………………………

………………………………………………………………………………………

………………………………………………………………………………………

18. What are the main impacts on you (or your family) of not having enough food to eat on a regular basis?

…………………………………………………………………………………………

…………………………………………………………………………………………

…………………………………………………………………………………………

…………………………………………………………………………………………

…………………………………………………………………………………………

…………………………………………………………………………………………

19. In the last week, which places did you get food (tick one or more boxes)

- ☐ Supermarket
- ☐ Deli / cafe / coffee shop
- ☐ Takeaway or fast food (e.g. Hungry Jacks, McDonalds, Pizza etc)
- ☐ Pub or restaurant
- ☐ Hostel
- ☐ Emergency accommodation
- ☐ Soup van, Doorway, Red Cross, Manna
- ☐ Other Church or welfare organisation
- ☐ Places that dump food (e.g. bins at the back of supermarkets or restaurants)
- ☐ Hospital
- ☐ Friends or relatives
- ☐ Other, please explain…………………………………………………………

…………………………………………………………………………………………

…………………………………………………………………………………………

20. Which of the following have you done to obtain food?

	Never	Sometimes	Often
Ask people on the street for food	☐	☐	☐
Ask people on the street for money	☐	☐	☐
Taken food from rubbish bins	☐	☐	☐
Stolen money to buy food	☐	☐	☐
Stolen food or drink	☐	☐	☐

21. How much do you usually spend on food each week? (tick one box)
- ☐ Nothing, **go to question 23**
- ☐ Less than $20
- ☐ $21-$50
- ☐ $51 -100
- ☐ More than $100 → How much? ……………………………………….

22. Where do you buy most of your food (tick one or more boxes)
- ☐ Pie van / cart
- ☐ Deli's / sandwich bars / cafes / coffee shop
- ☐ Supermarket
- ☐ Takeaway or fast food (e.g. Hungry Jacks, McDonalds, Pizza etc)
- ☐ Other, please describe:……………………………………………………

……………………………………………………………………………………

……………………………………………………………………………………

23. List three foods you would buy if you had an extra $20 a week to spend on food (please list below)

1. ………………………………………………………………………………
2. ………………………………………………………………………………
3. ………………………………………………………………………………

24. How long have you been getting food from charitable food services?
- ☐ This is the first time
- ☐ Days
- ☐ Weeks
- ☐ Months
- ☐ A year
- ☐ More than a year, (how many years?______)

25. What do you think would improve charitable food services?

……………………………………………………………………………………

……………………………………………………………………………………

……………………………………………………………………………………

……………………………………………………………………………………

……………………………………………………………………………………

……………………………………………………………………………………

……………………………………………………………………………………

……………………………………………………………………………………

26. How important is it that charitable food services provide the following?

	Unimportant	Neither Important or unimportant	Important
Water	☐	☐	☐
Coffee / tea	☐	☐	☐
Soup	☐	☐	☐
Meat pies	☐	☐	☐
Sweets and biscuits	☐	☐	☐
Healthy food	☐	☐	☐
Cooked meals	☐	☐	☐
Vegetarian food	☐	☐	☐
Foods for different cultures	☐	☐	☐
Soft foods that don’t need much chewing	☐	☐	☐
A place to sit and eat with others	☐	☐	☐
Food and drinks at the right temperature	☐	☐	☐
Knives, forks, spoons	☐	☐	☐

27. Have you used any of the following services?

	Yes	No
Money management or budgeting	☐	☐
Employment assistance	☐	☐
Accommodation assistance	☐	☐
Transport assistance	☐	☐
General health services	☐	☐
Drug and alcohol services	☐	☐
Reskilling / access to education	☐	☐

28. Are there any other services the charitable food services should provide?

…….…………………………………………………………………………………………

……………..……………………………………………………………………………

………………………..…………………………………………………………………

……………………………..……………………………………………………………

29. In general, which best describes your overall health? (tick one box)

- ☐ Excellent
- ☐ Good
- ☐ Poor

30. In general, would you say your teeth are? (tick one box)

- ☐ Excellent
- ☐ Good
- ☐ Poor

31. When was the last time you saw a dentist?

- ☐ Last year
- ☐ 1-2 years ago
- ☐ More than 2 years ago

32. When was the last time you saw a doctor?

- ☐ Last week
- ☐ Last month
- ☐ Last year
- ☐ 1-2 years ago
- ☐ More than 2 years ago, how many years? _____

33. Has a doctor said you have any of the following? (tick all that apply)

- ☐ Diabetes
- ☐ Heart disease
- ☐ Mental health issues
- ☐ Cancer
- ☐ Asthma or other chronic lung illness
- ☐ Other, please describe:…………………………………………………...

…………..………………………………………………………………………………

34. In the past 3 months have you?

- ☐ Gained weight
- ☐ Lost weight
- ☐ Neither gained or lost
- ☐ Don’t know.

35. Where do you get most of your income from? (tick all that apply)

- ☐ Youth Allowance Dependent Rate
- ☐ Youth Allowance Independent Rate
- ☐ Disability Support Pension
- ☐ Sickness Benefits
- ☐ Parenting Allowance
- ☐ Partner Allowance
- ☐ Friends or family
- ☐ Wages / Job
- ☐ Other, please describe:……………………………………………………..

………..………………………………………………………………………………

36. How much money do you usually get a fortnight?

$............................ per fortnight

☐ Don't know

37. In terms of housing, do you?

- ☐ Rent
- ☐ Have a mortgage
- ☐ Own your home
- ☐ Live in temporary accommodation
- ☐ Live on the street.

38. Where have you slept in the last month? (tick all that apply)

- ☐ Crisis accommodation
- ☐ Friend's house
- ☐ Squats
- ☐ Backpackers
- ☐ Hostel
- ☐ Night shelter
- ☐ Private rental
- ☐ Outdoors / streets / sleeping rough / park
- ☐ Car
- ☐ Detention or lock-up
- ☐ Extended family or relatives
- ☐ My own house
- ☐ Other, please describe:…………………………………………..……..

………..………………………………………………………………………………

Thank you for your time.

OFFICE USE ONLY

Survey venue: ____

Day and date: ____ / _____/ 2016

Height: _______________ (cm) Weight: _______________ (kilograms)
